# Supplementary material for: Vernonanthura tweediana (Baker) H. Rob. (Asteraceae), an Ordinary Bush or an Anti-Inflammatory and Immunomodulator Aromatic Species?
Source: Pharmaceuticals (Basel). 2024 Nov 7;17(11):1492. doi: 10.3390/ph17111492 (PMC11597521; doi:10.3390/ph17111492)
Supplement: Supplementary file 1 [file pharmaceuticals-17-01492-s001.zip › pharmaceuticals-3259111-supplementary.pdf]

Supplementary Material

Table of Contents

Figure S1 – GC-FID chromatogram of the essential oil of dry leaves from *Vernonanthura tweediana*, Pomerode, SC-Brazil 2

Figure S2 – GC-MS chromatogram of the essential oil of dry leaves from *Vernonanthura tweediana*, Pomerode, SC-Brazil 3

Figure S3 – A. Expanded GC-MS chromatogram of the essential oil from dried leaves from *Vernonanthura tweediana* (4.65 – 18.80 min). Identified peaks are assigned with the entries 1-14; **B**. Expanded GC-MS chromatogram of the essential oil of dry leaves from *Vernonanthura tweediana* (19.59 – 25.95 min). Identified peaks are assigned with the entries 15-27; **C**. Expanded GC-MS chromatogram of the essential oil of dry leaves from *Vernonanthura tweediana* (27.20 – 47.88 min). Identified peaks are assigned with the entries 28 – 36 4

MS Data …………………………………………………………………………………………………... 6

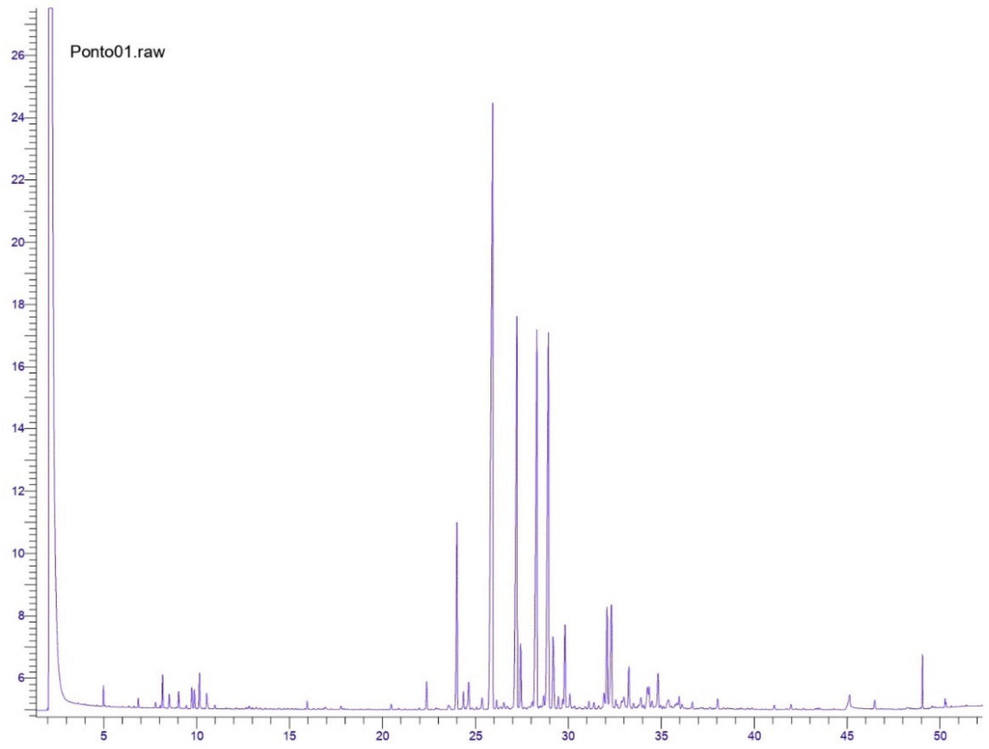

Figure S1 – GC-FID chromatogram of the essential oil of dry leaves from *Vernonanthura tweediana*, Pomerode, SC-Brazil.

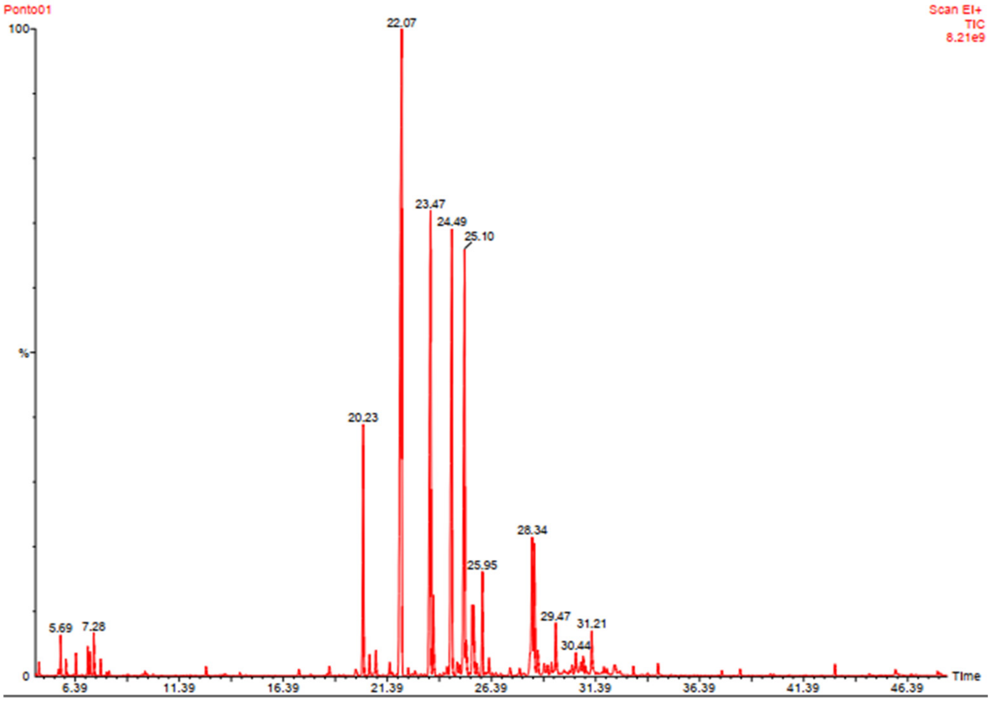

Figure S2 – GC-MS chromatogram of the essential oil of dry leaves from *Vernonanthura tweediana*, Pomerode, SC-Brazil.

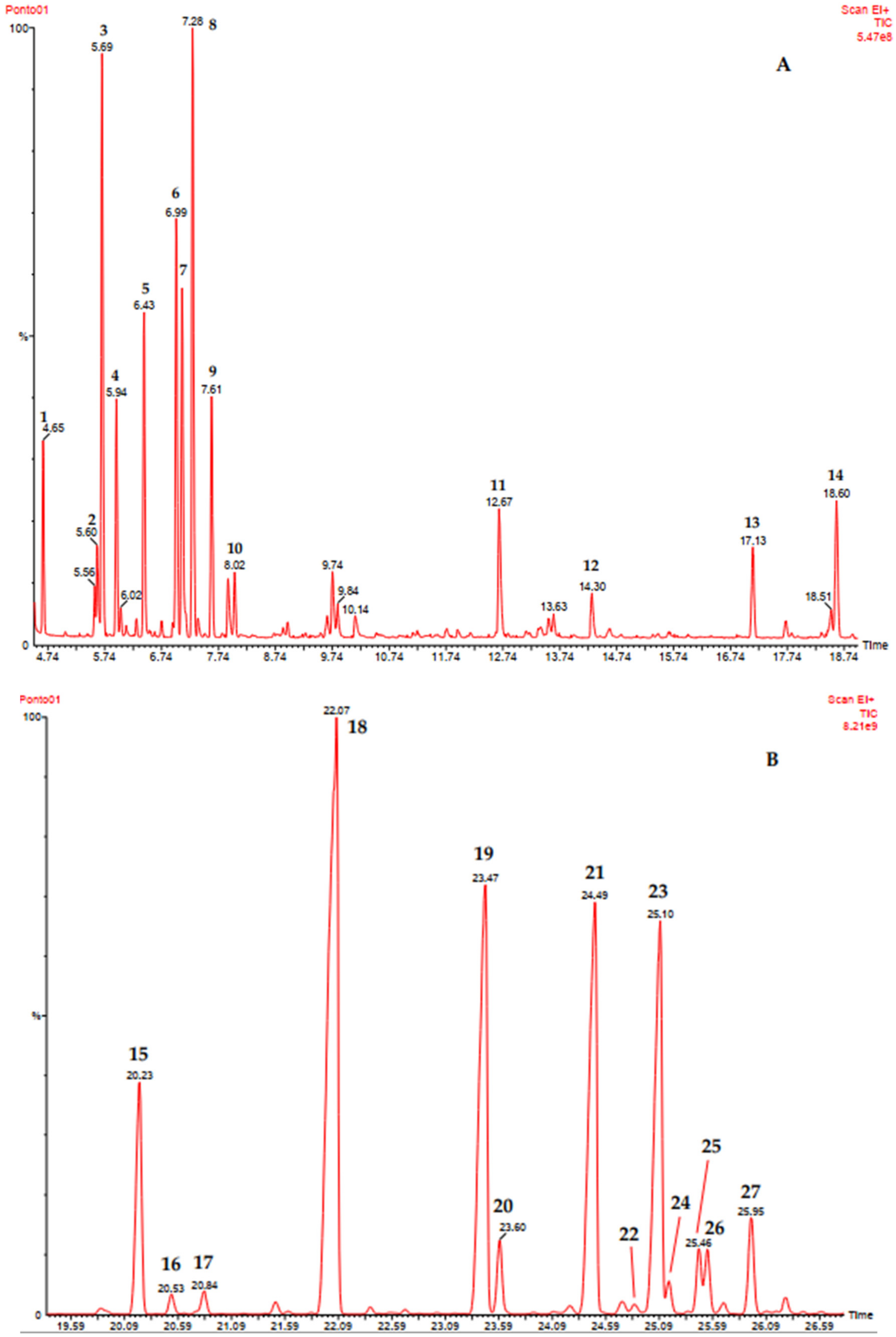
Ponto01
Scan EI+
TIC
5.47e8
A
100
%
0
Time
4.74
5.74
6.74
7.74
8.74
9.74
10.74
11.74
12.74
13.74
14.74
15.74
16.74
17.74
18.74
1 4.65
2 5.60
5.56
3 5.69
4 5.94
6.02
5 6.43
6 6.99
7
7.28 8
9 7.61
10 8.02
9.74
9.84
10.14
11 12.67
13.63
12 14.30
13 17.13
18.51
14 18.60
Ponto01
Scan EI+
TIC
8.21e9
B
19.59
20.09
20.59
21.09
21.59
22.09
22.59
23.09
23.59
24.09
24.59
25.09
25.59
26.09
26.59
15 20.23
16 20.53
17 20.84
22.07 18
19 23.47
20 23.60
21 24.49
22
23 25.10
24
25
25.46
26
27 25.95

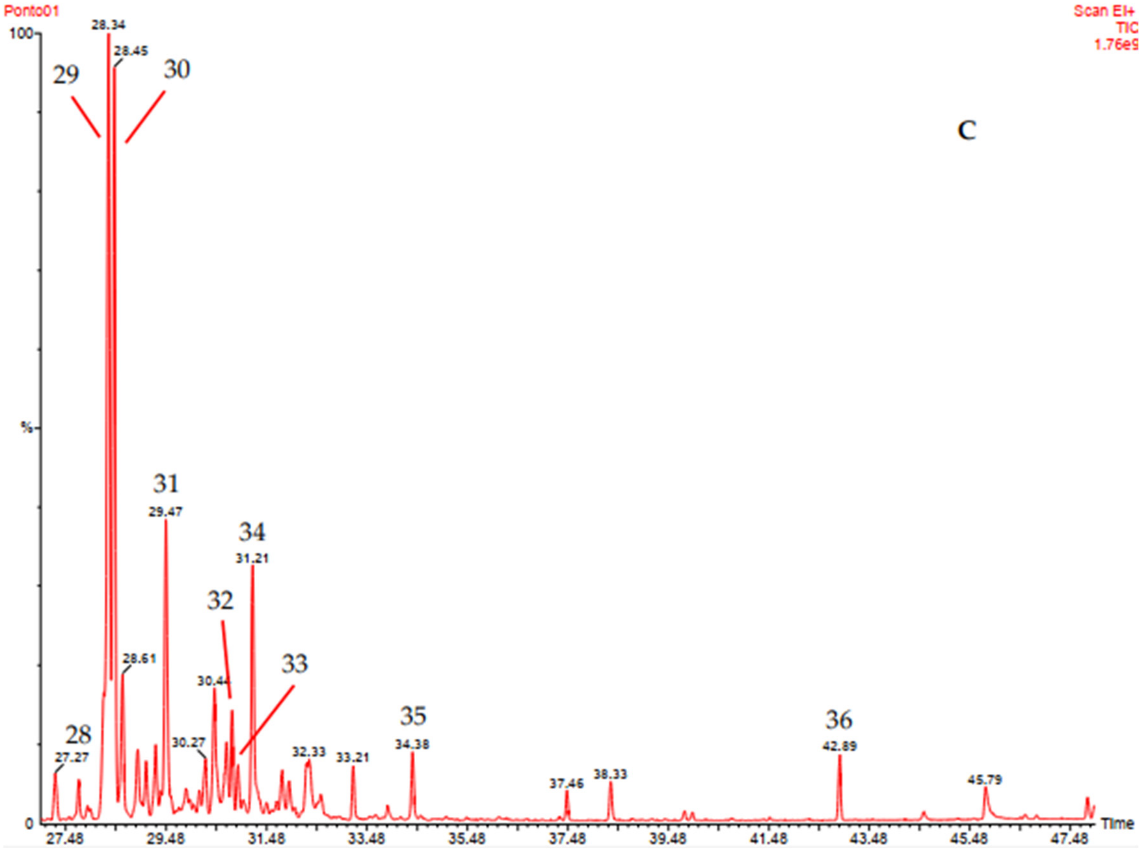

Figure S3 – A. Expanded GC-MS chromatogram of the essential oil from dried leaves from *Vernonanthura tweediana* (4.65 – 18.80 min). Identified peaks are assigned with the entries 1-14; **B**. Expanded GC-MS chromatogram of the essential oil of dry leaves from *Vernonanthura tweediana* (19.59 – 25.95 min). Identified peaks are assigned with the entries 15-27; **C**. Expanded GC-MS chromatogram of the essential oil of dry leaves from *Vernonanthura tweediana* (27.20 – 47.88 min). Identified peaks are assigned with the entries 28 – 36.

MS Data - Spectra of the constituents and the MS spectra of the identified compound from the NIST library. For each spectrum, the two main hits are given.

Entry 1: α-pinene.

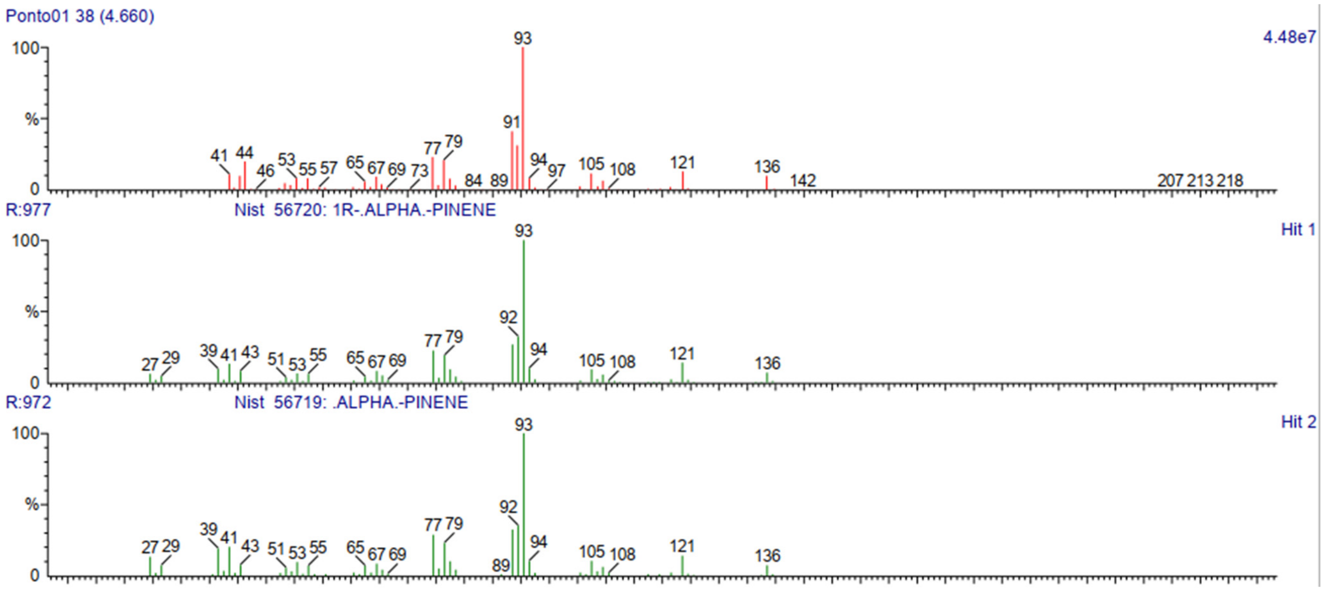

Entry 2: benzaldehyde.

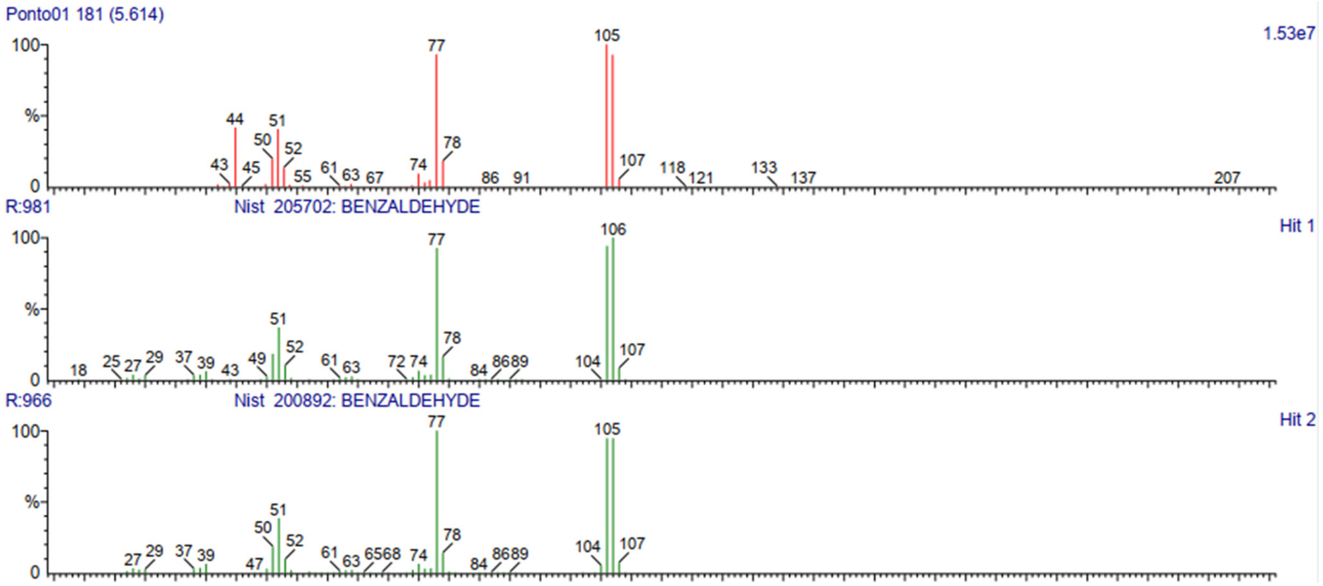

Entry 3: β-pinene.

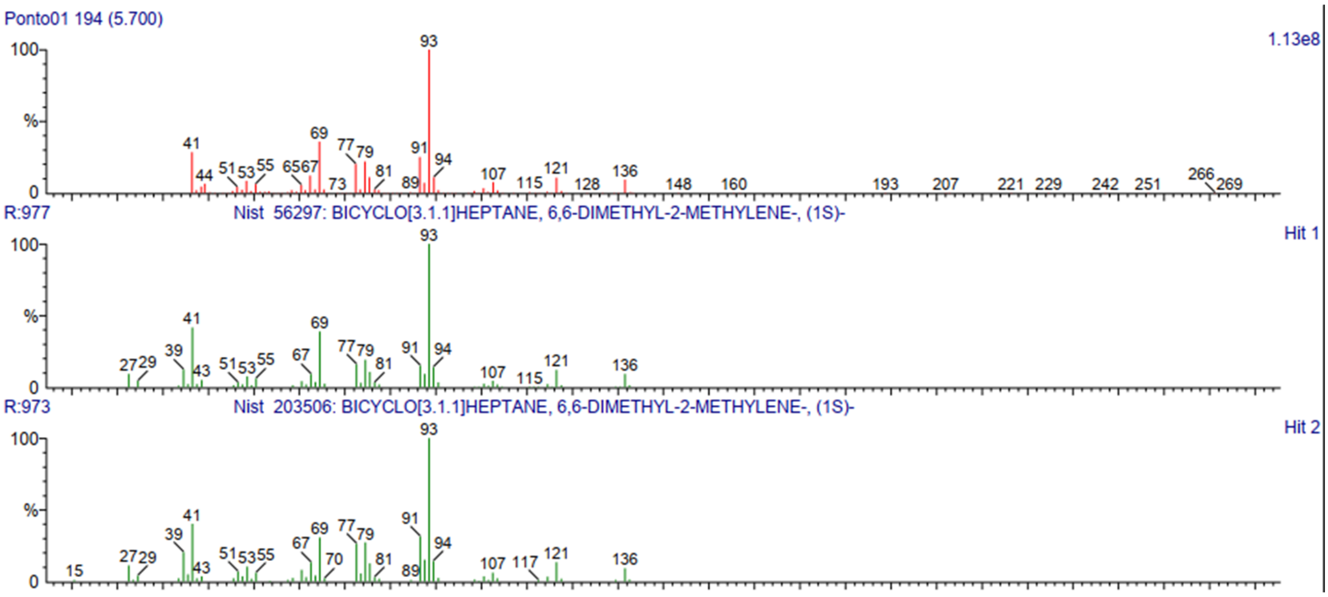

Entry 4: β-myrcene.

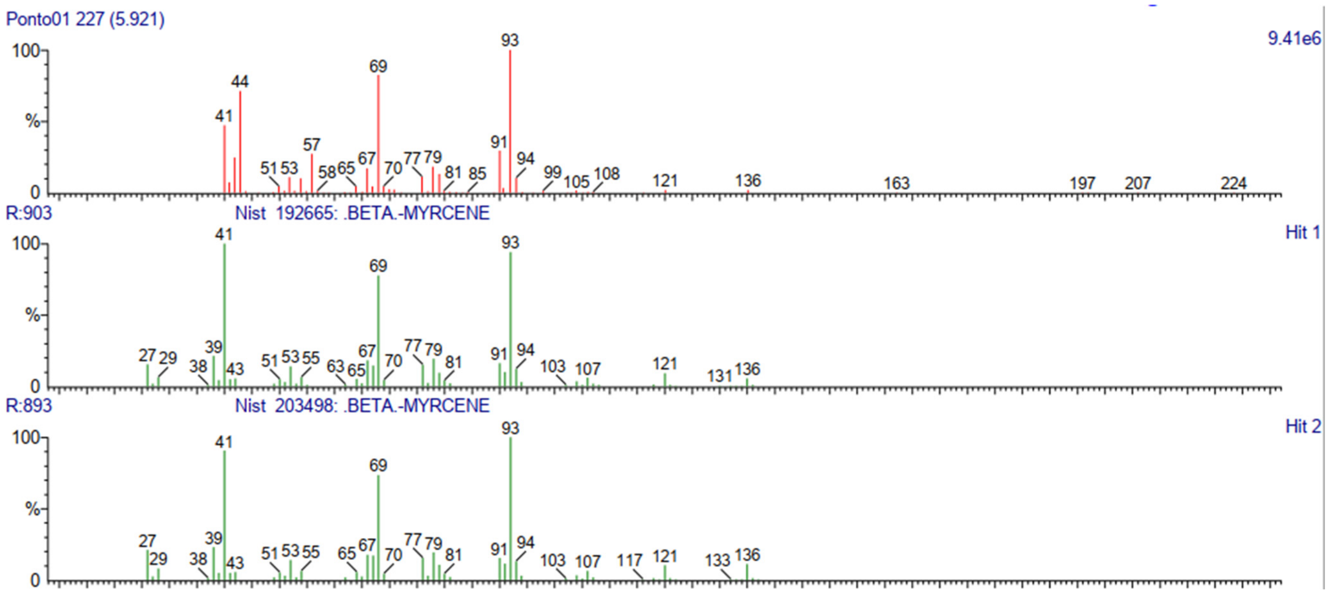

Entry 5: α-phellandrene.

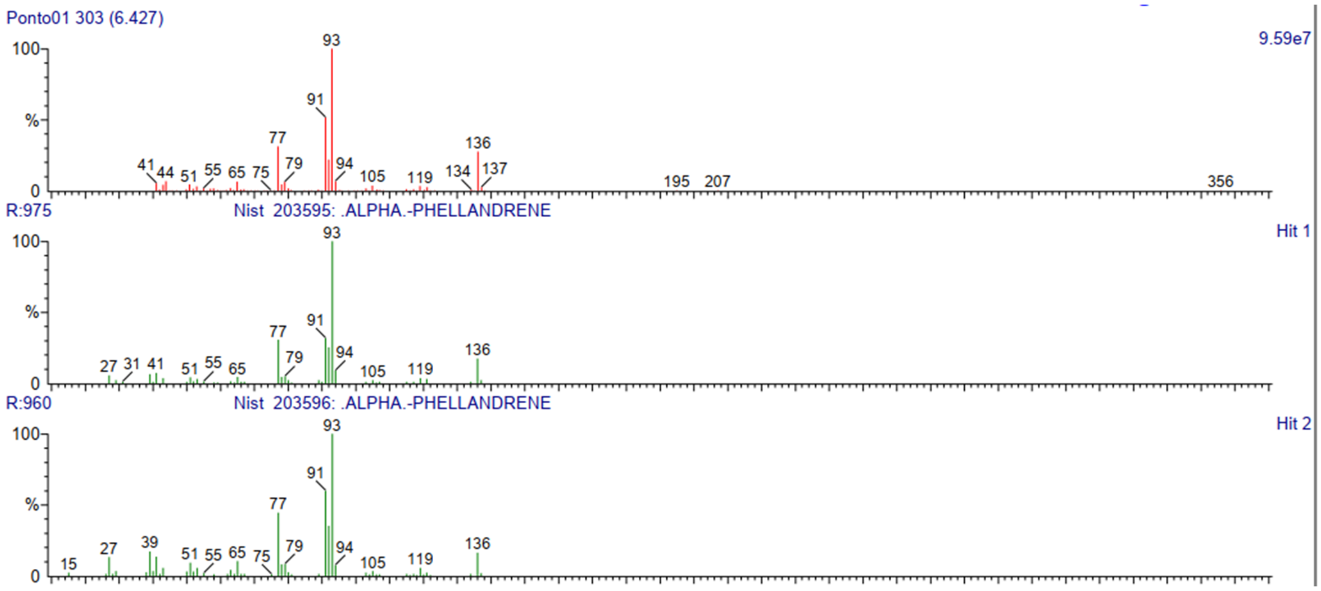

Entry 6: *ortho* or *para*-cymene.

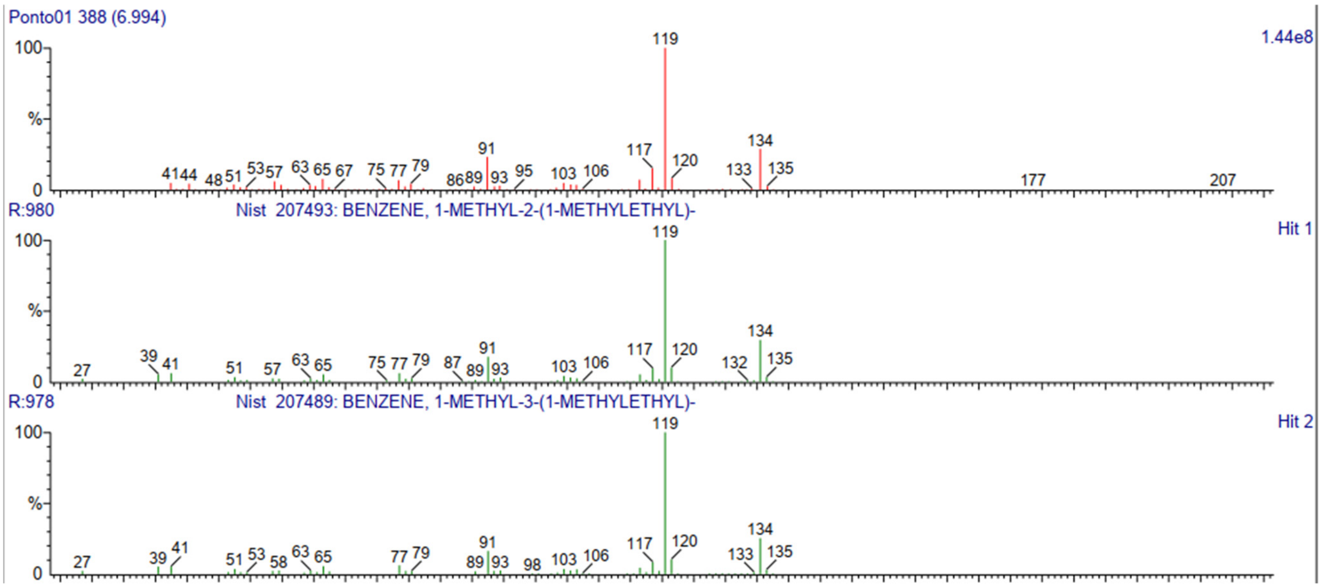

Entry 7: limonene.

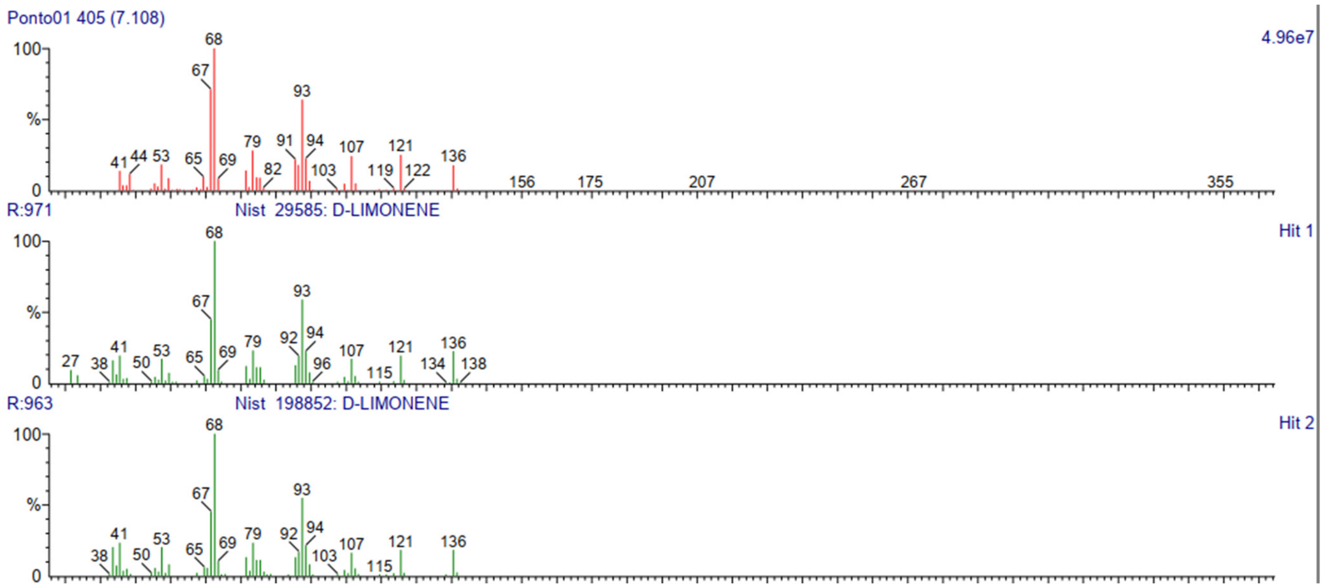

Entry 8: *Z*-β-ocimene ($AI_{lit.}$ 1032).

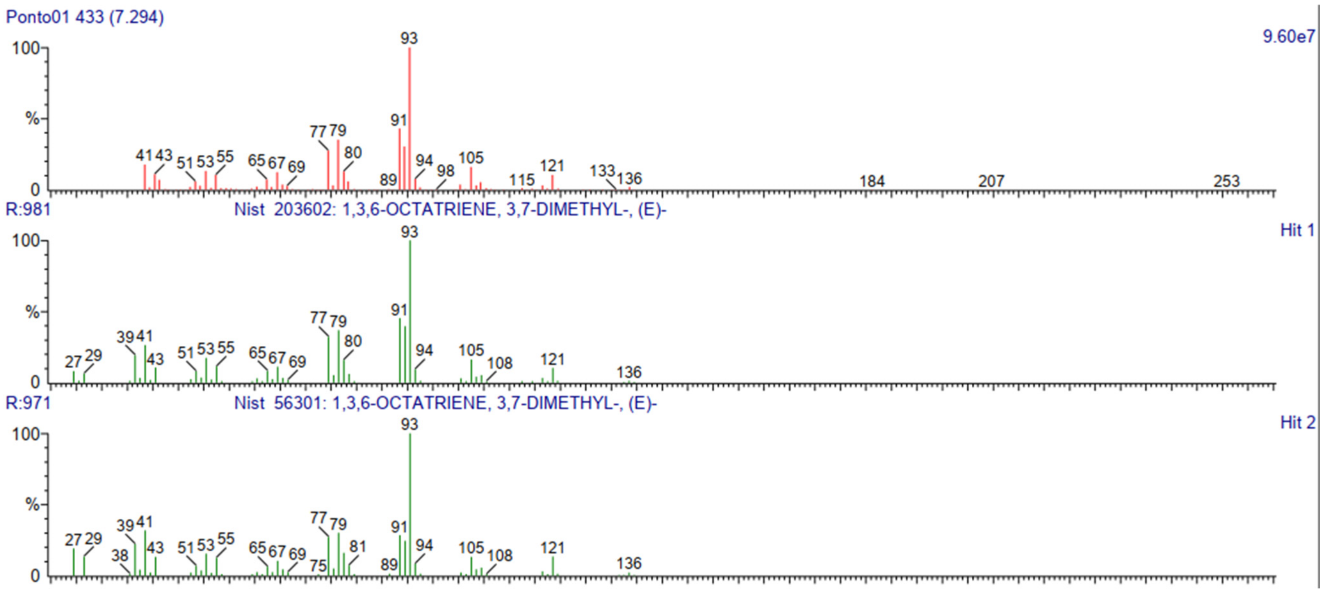

Entry 9: *E*-β-ocimene (AI$_{lit.}$ 1044).

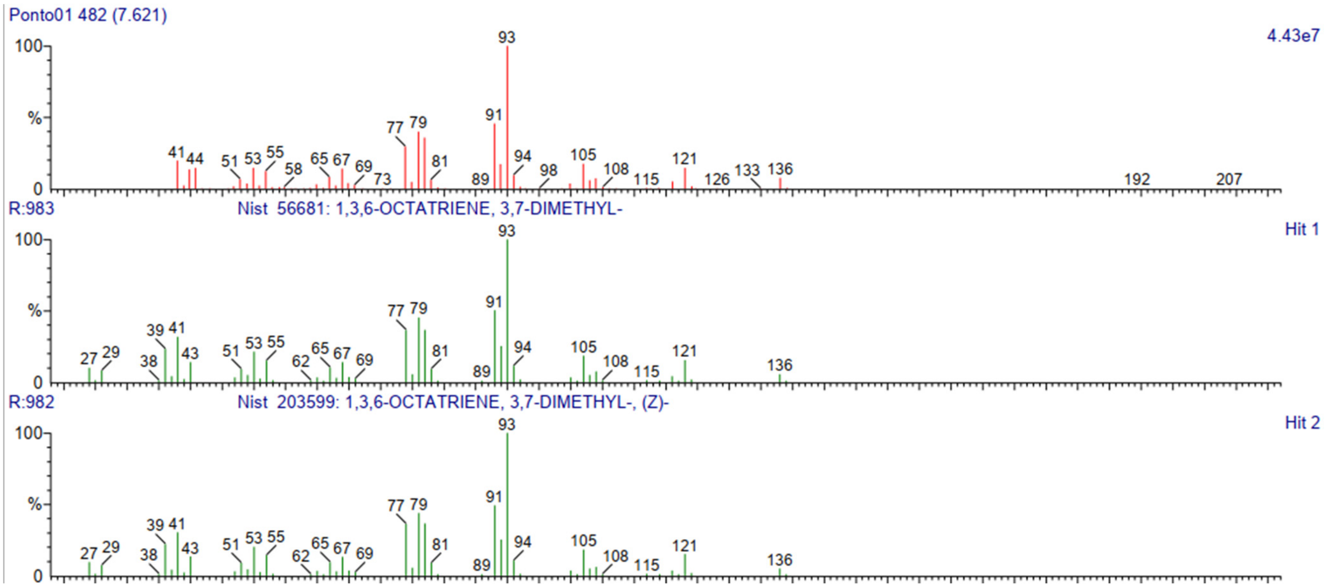

Entry 10: γ-terpinene.

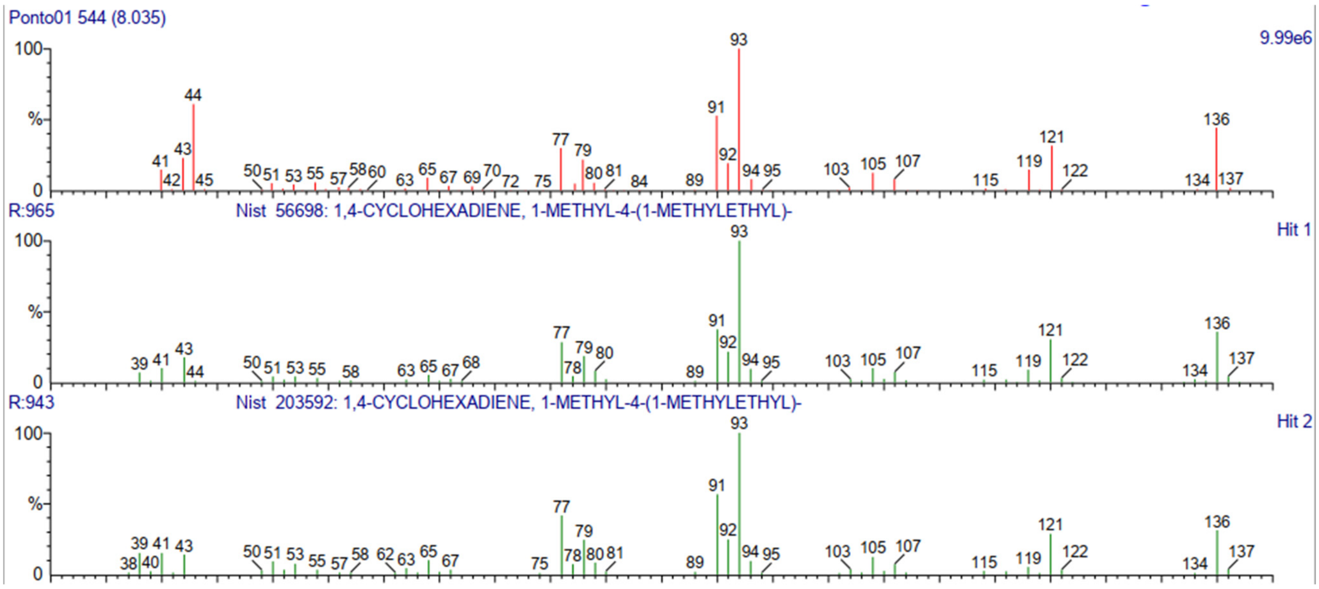

Entry 11: terpinen-4-ol.

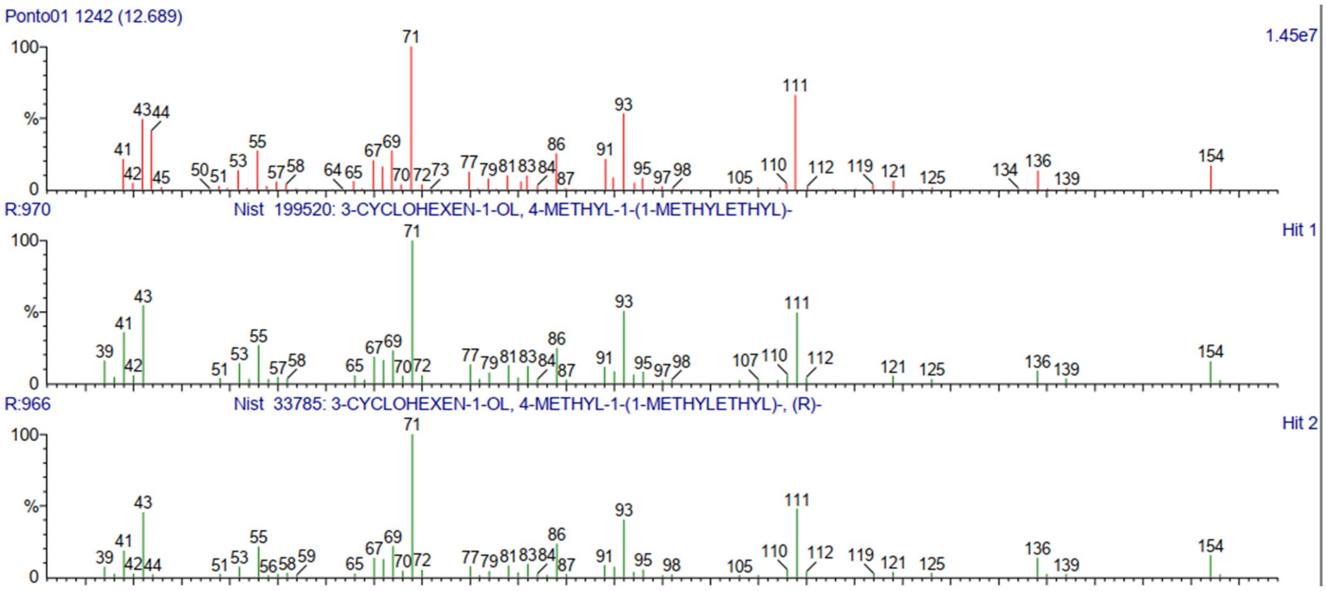

Entry 12: β-cyclocitral.

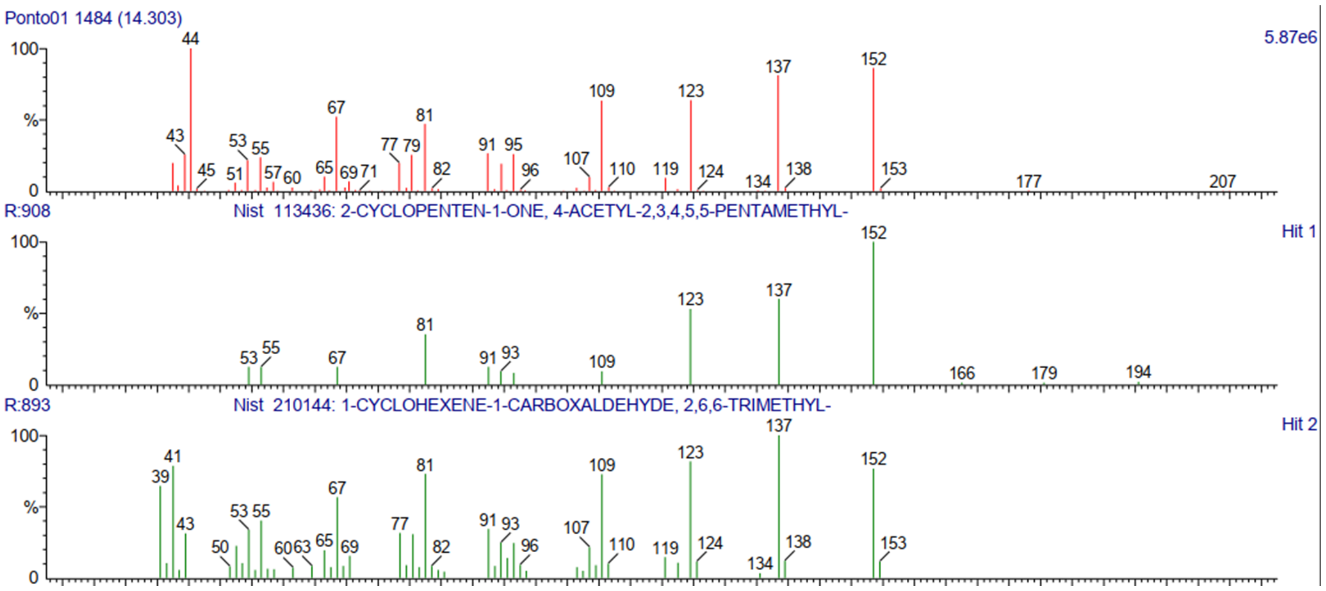

Entry 13: safrole.

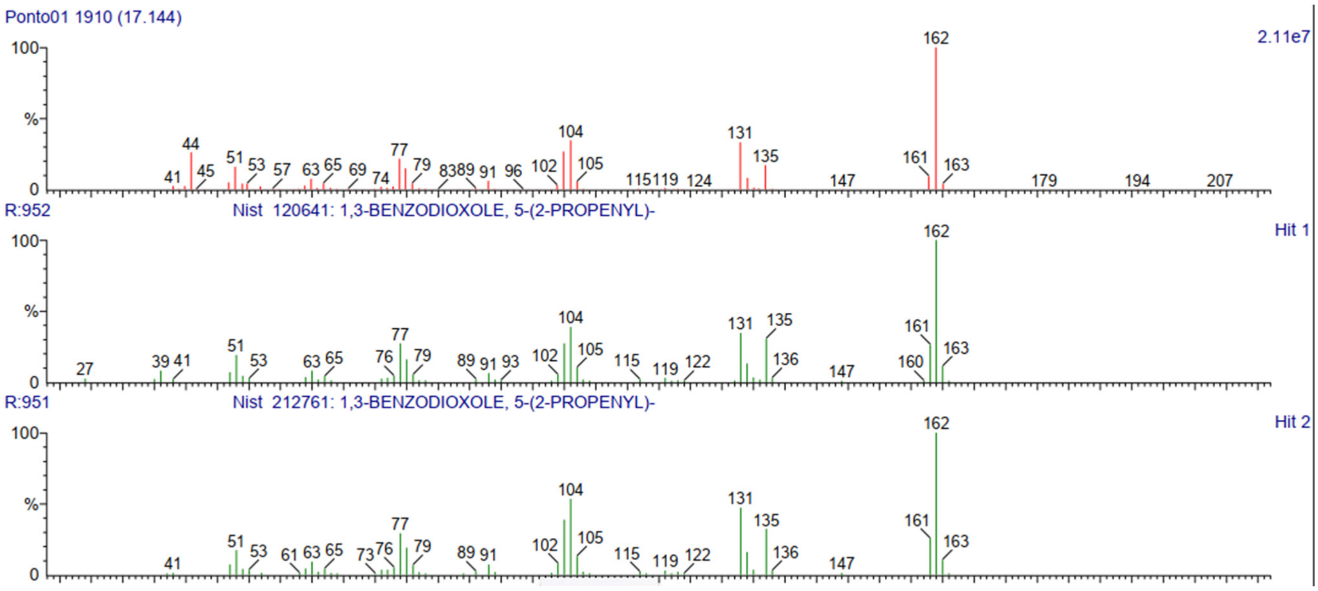

Entry 14: δ-elemene.

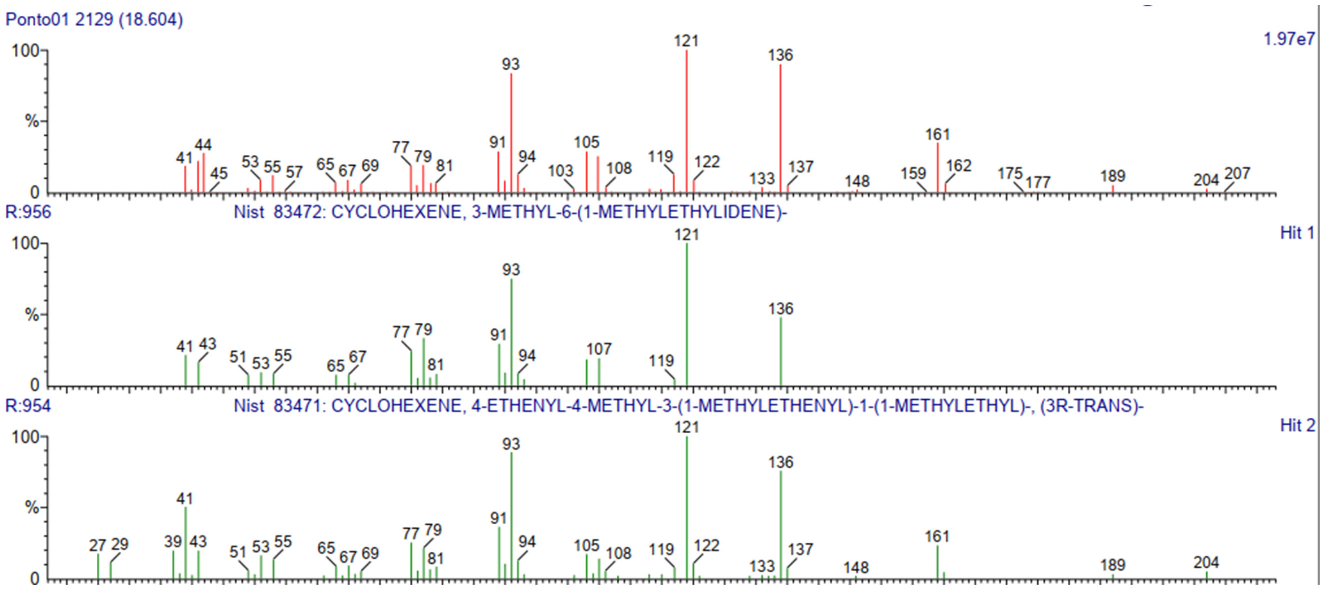

Entry 15: α-copaene.

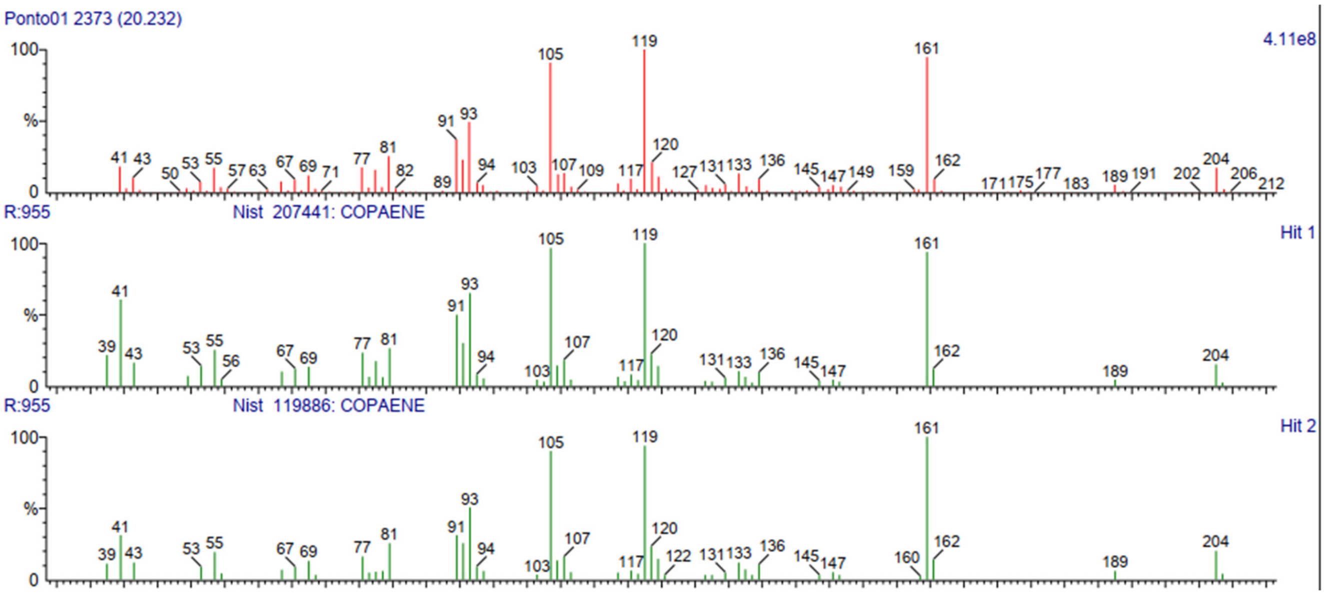

Entry 16: β-bourbonene.

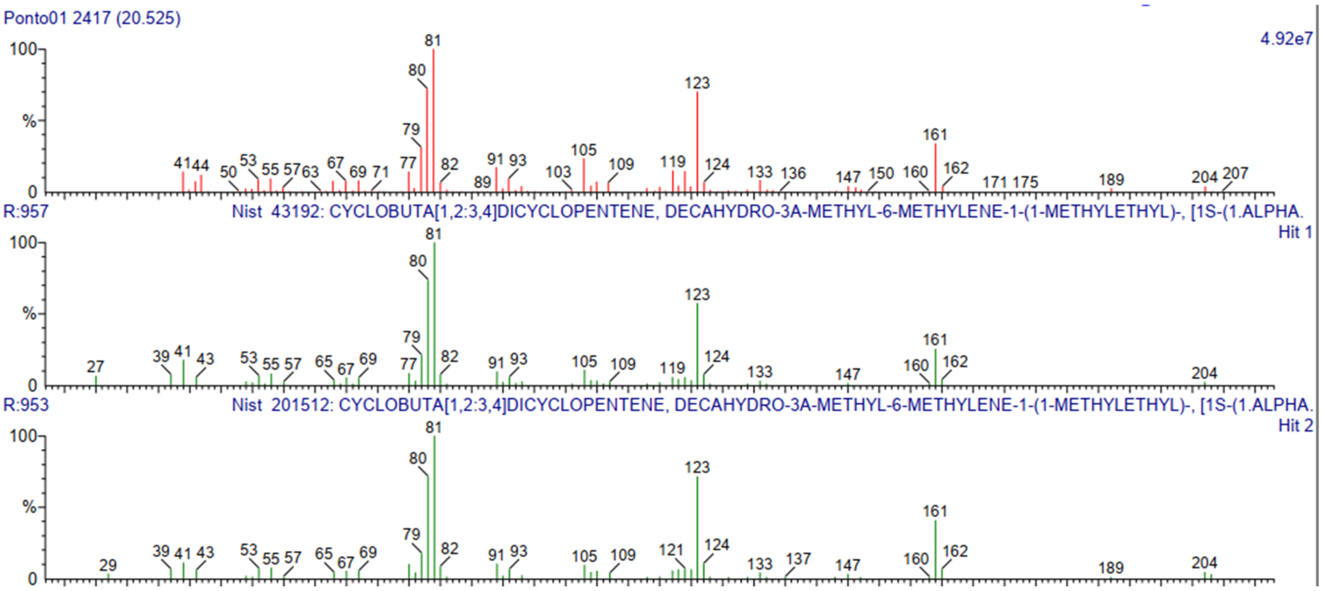

Entry 17: β-elemene.

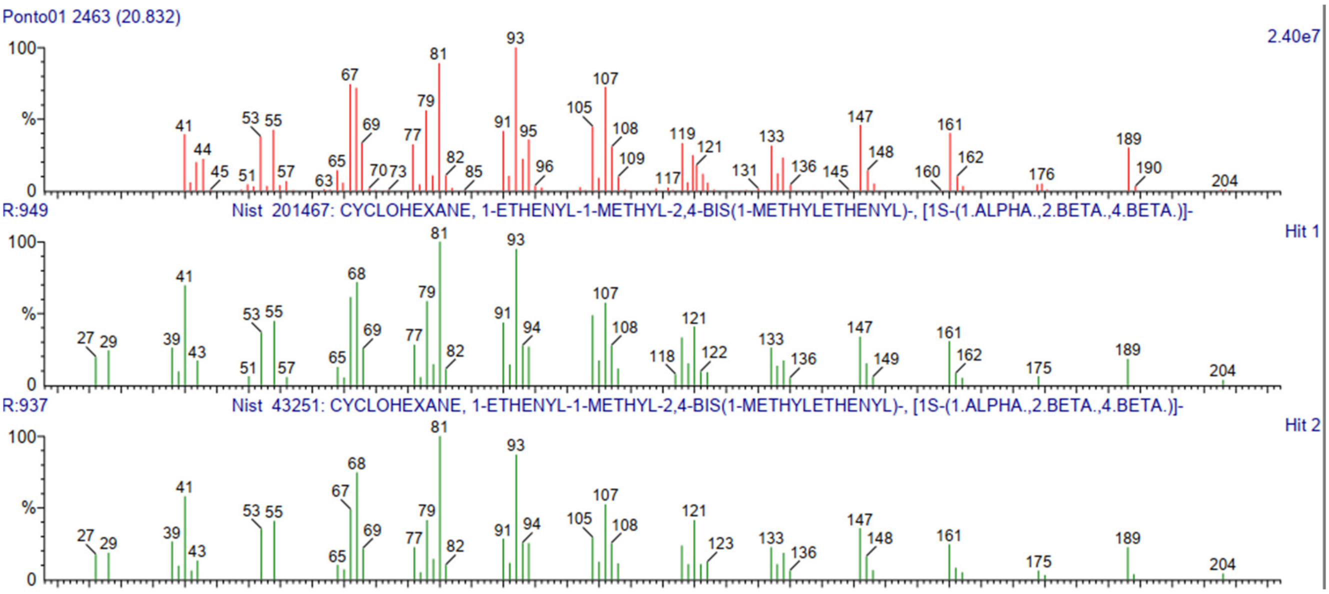

Entry 18: β-caryophyllene.

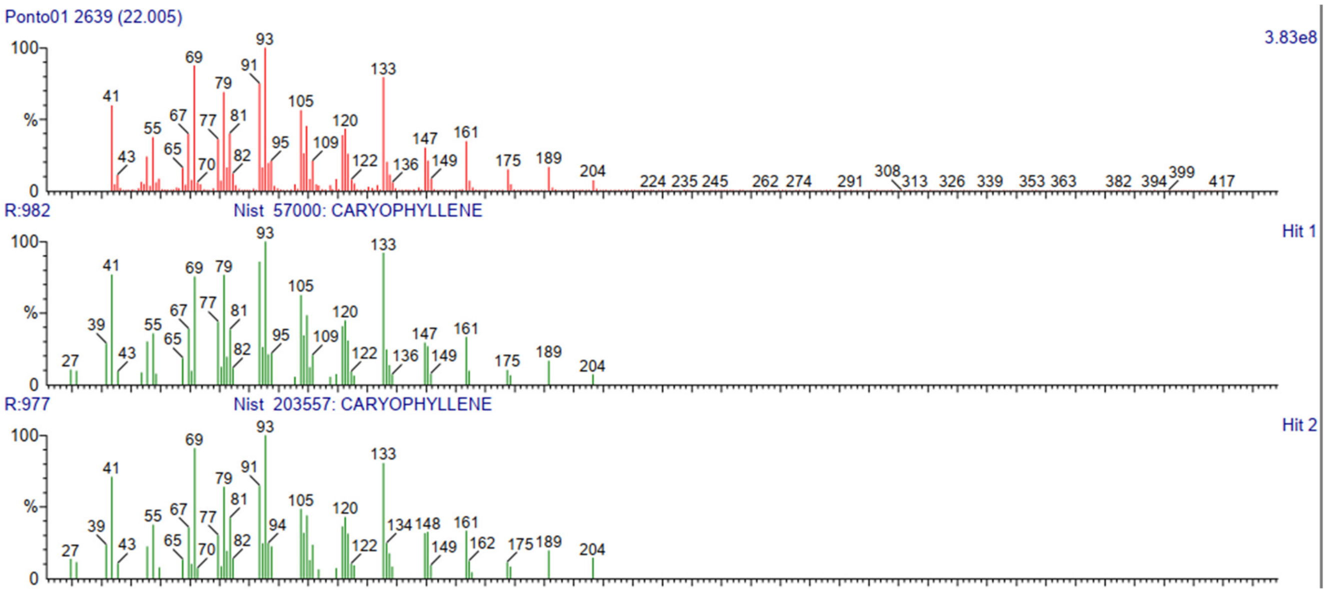

Entry 19: α-caryophyllene.

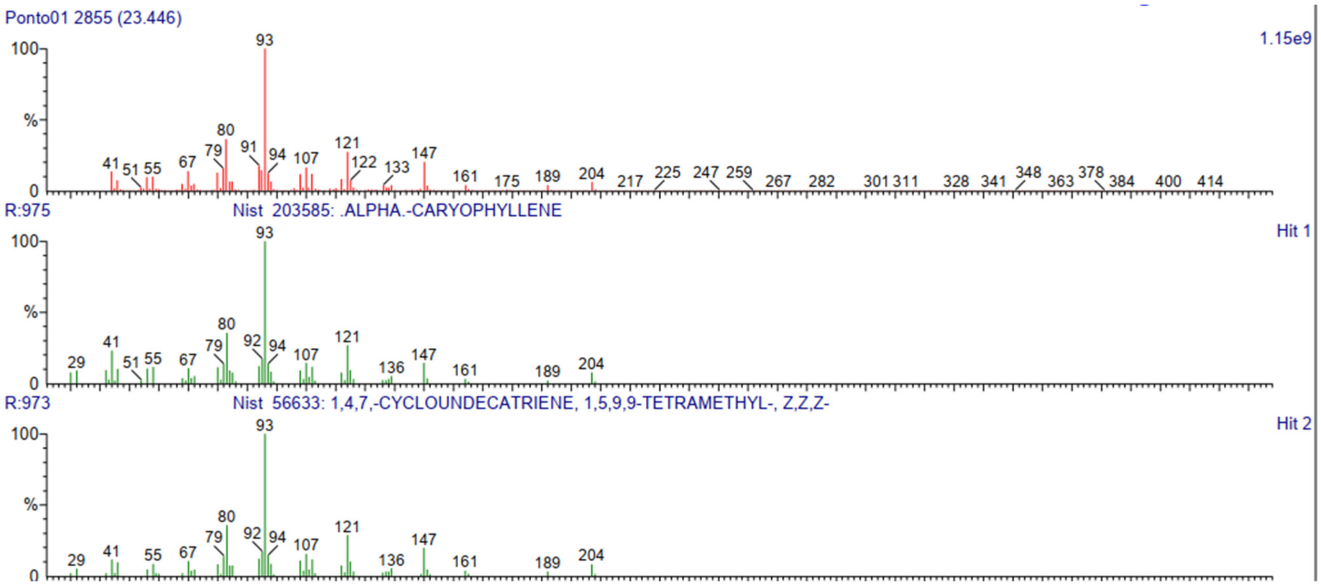

Entry 20: *allo*-aromadendrene.

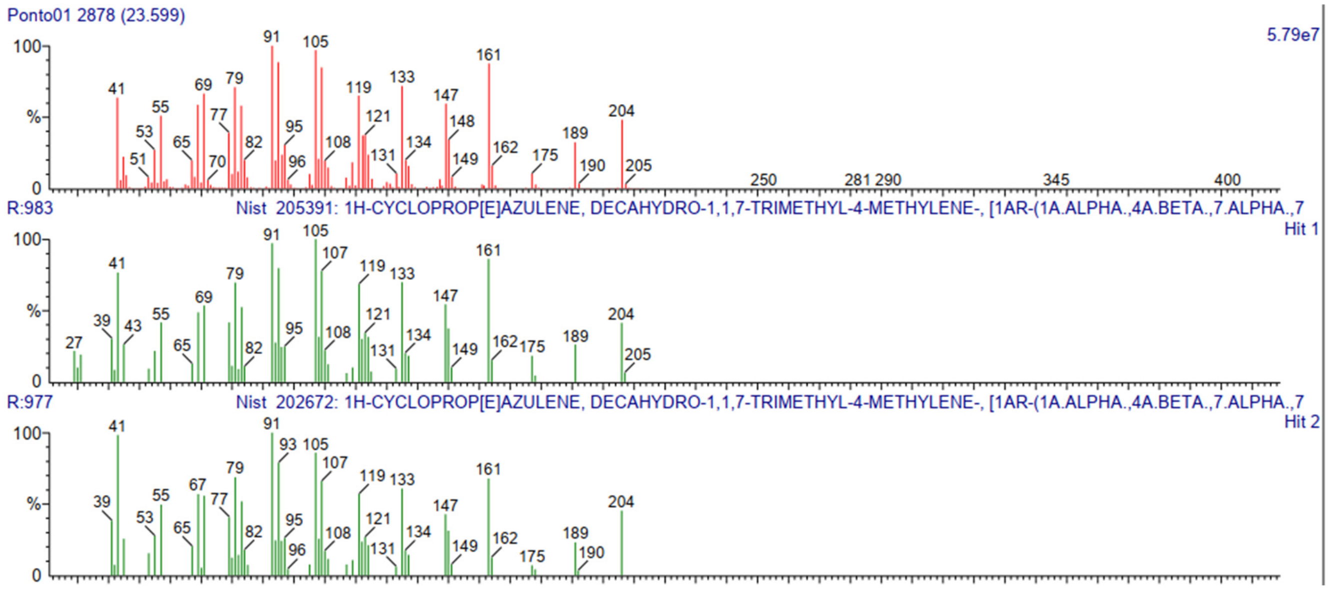

Entry 21: germacrene D.

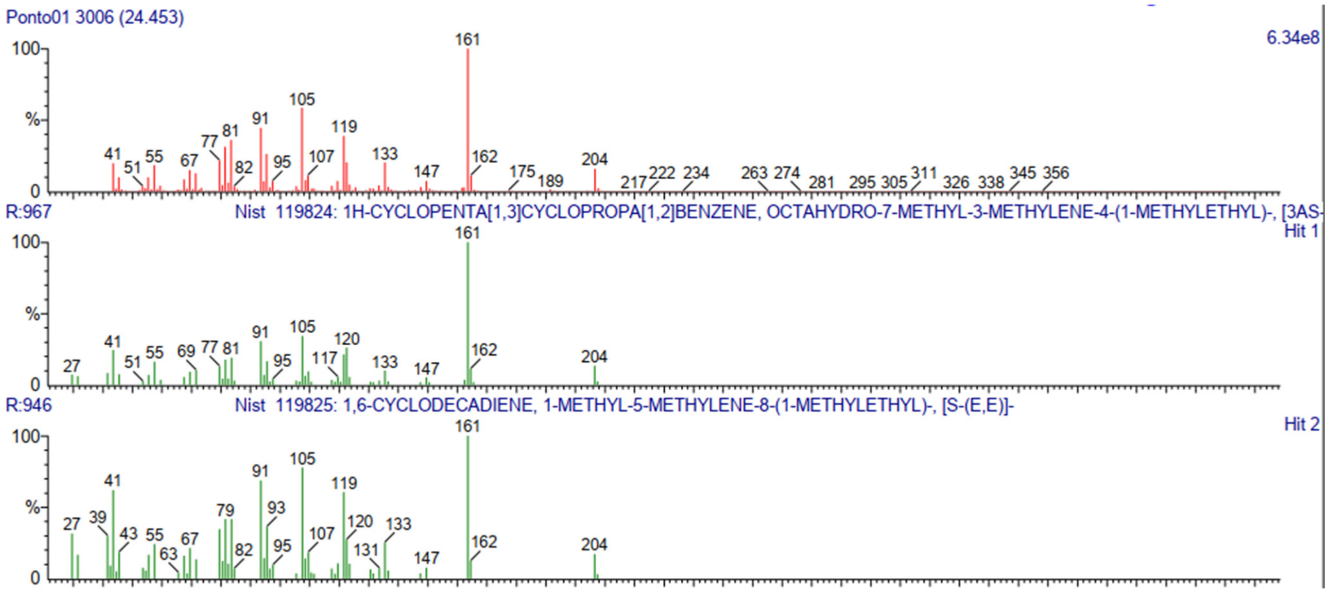

Entry 22: *E*-β-ionone.

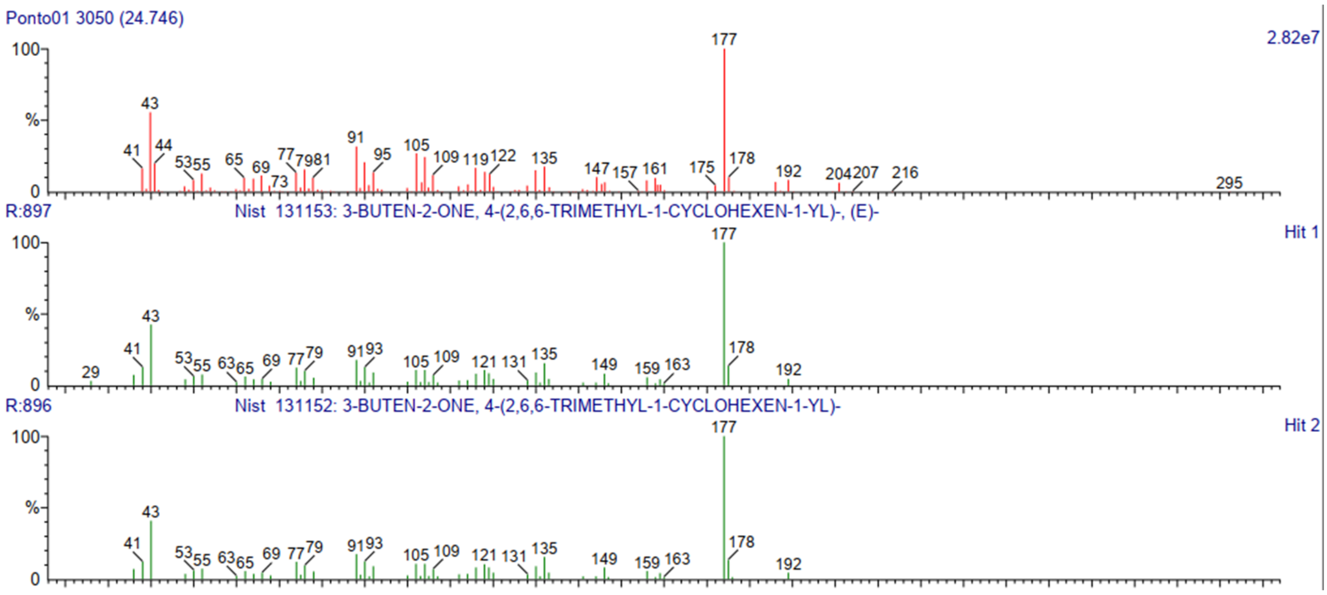

Entry 23: bicyclogermacrene. The identification was based on AI and literature [16].

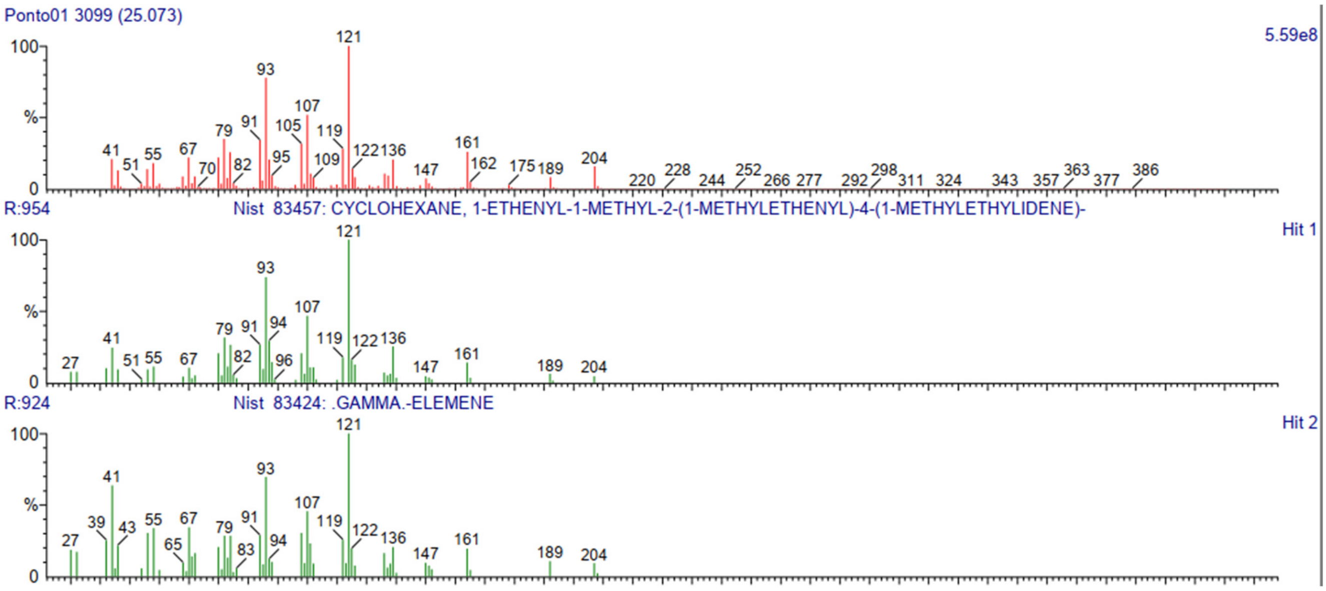

Entry 24: α-muurolene.

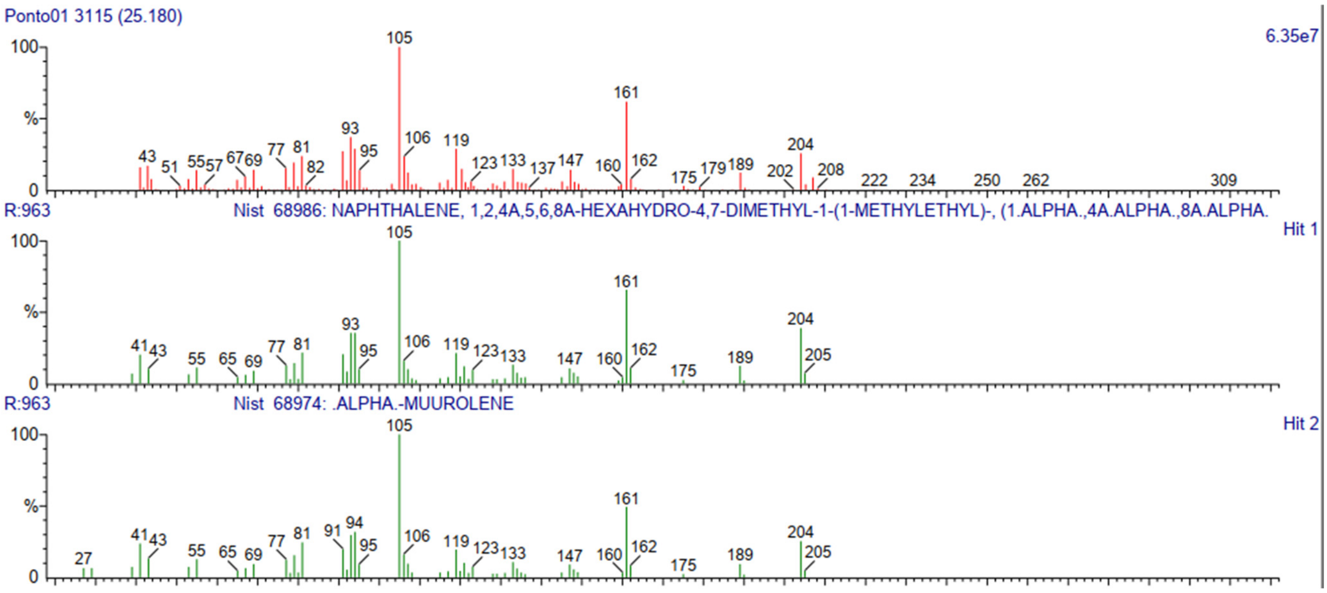

Entry 25: *E*,*E*-α-farnesene.

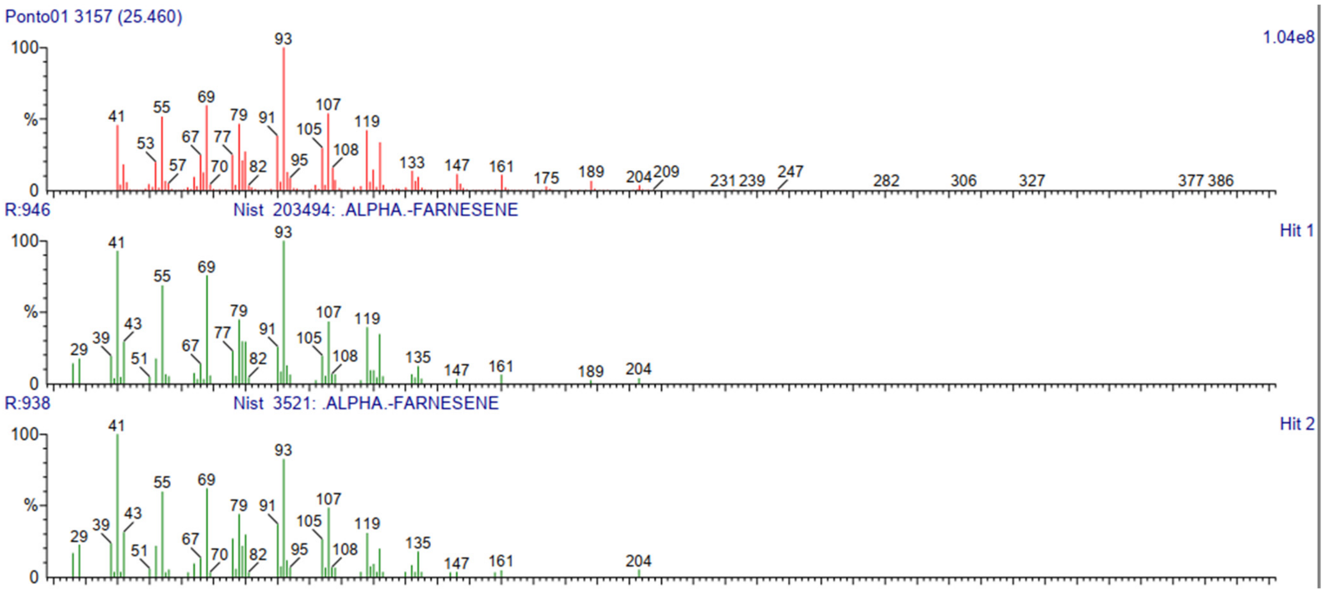

Entry 26: β-bisabolene.

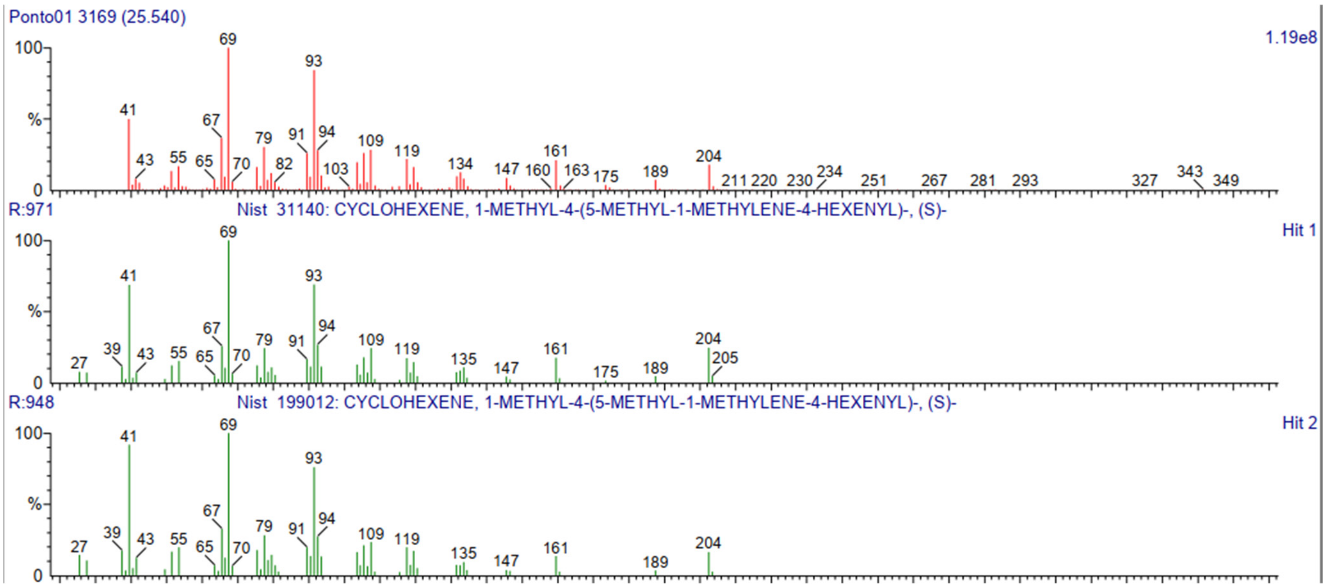

Entry 27: δ-cadinene.

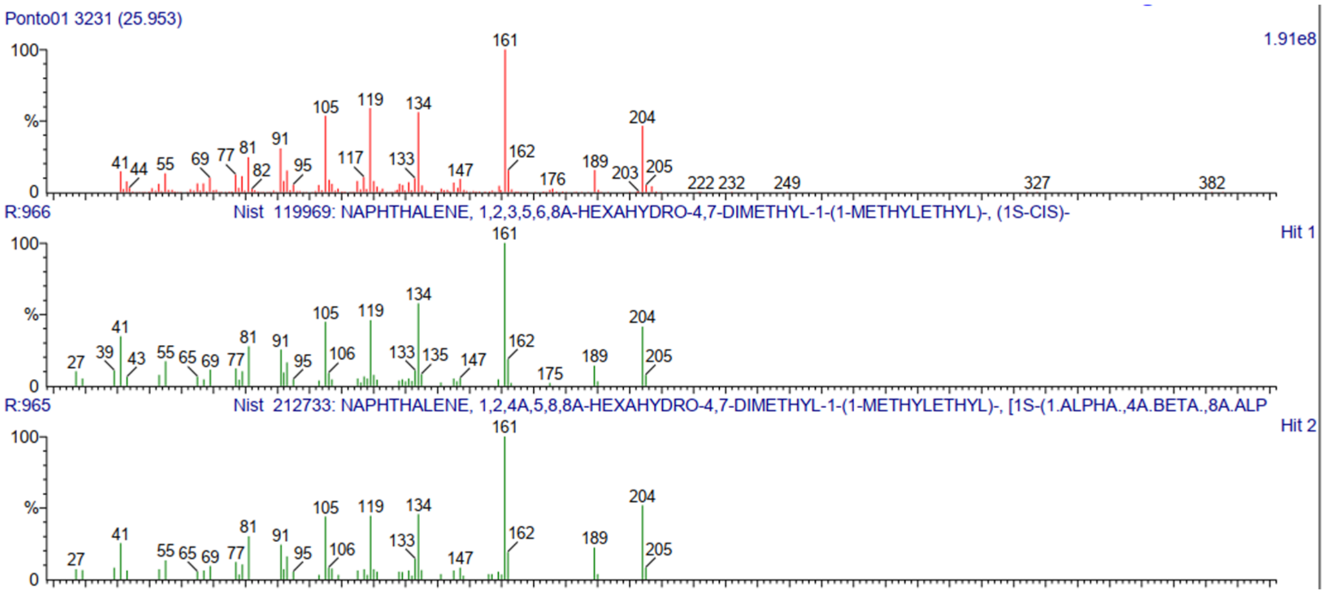

Entry 28: *E*-nerolidol.

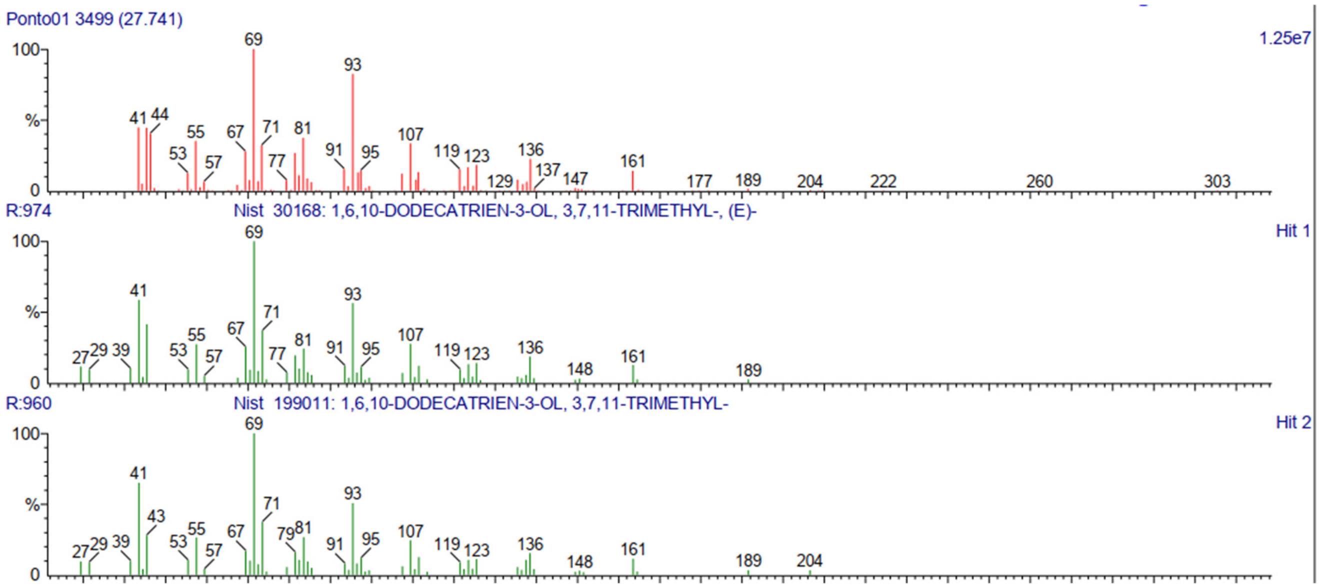

Entry 29: spathulenol.

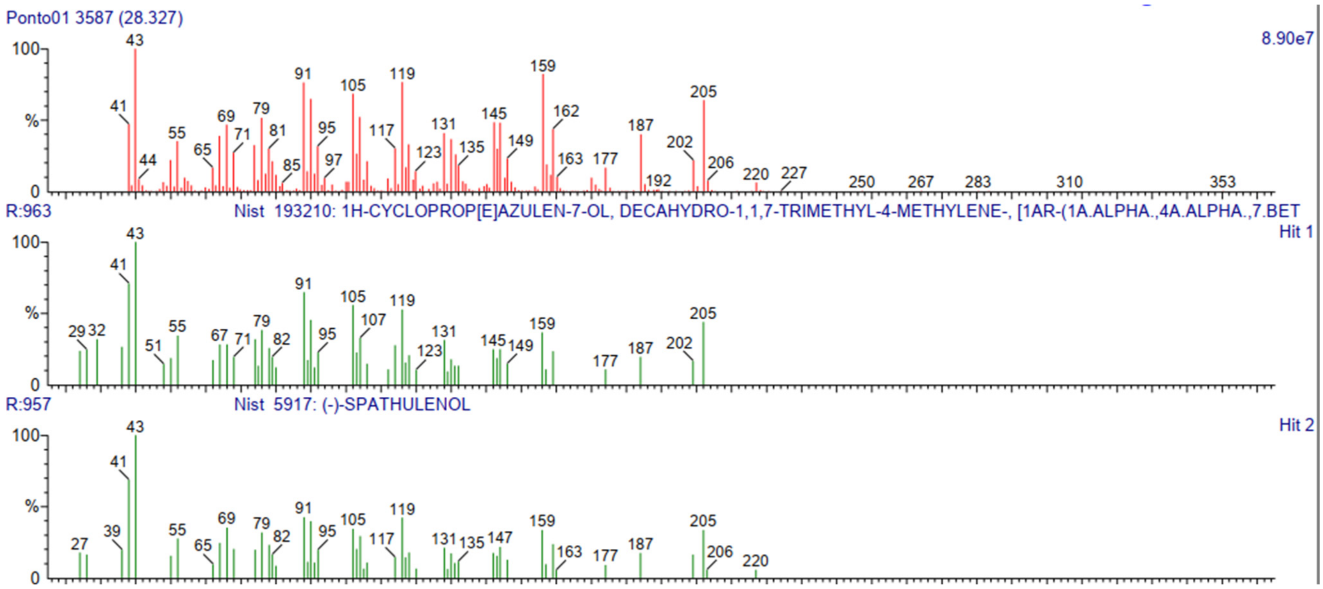

Entry 30: caryophyllene oxide.

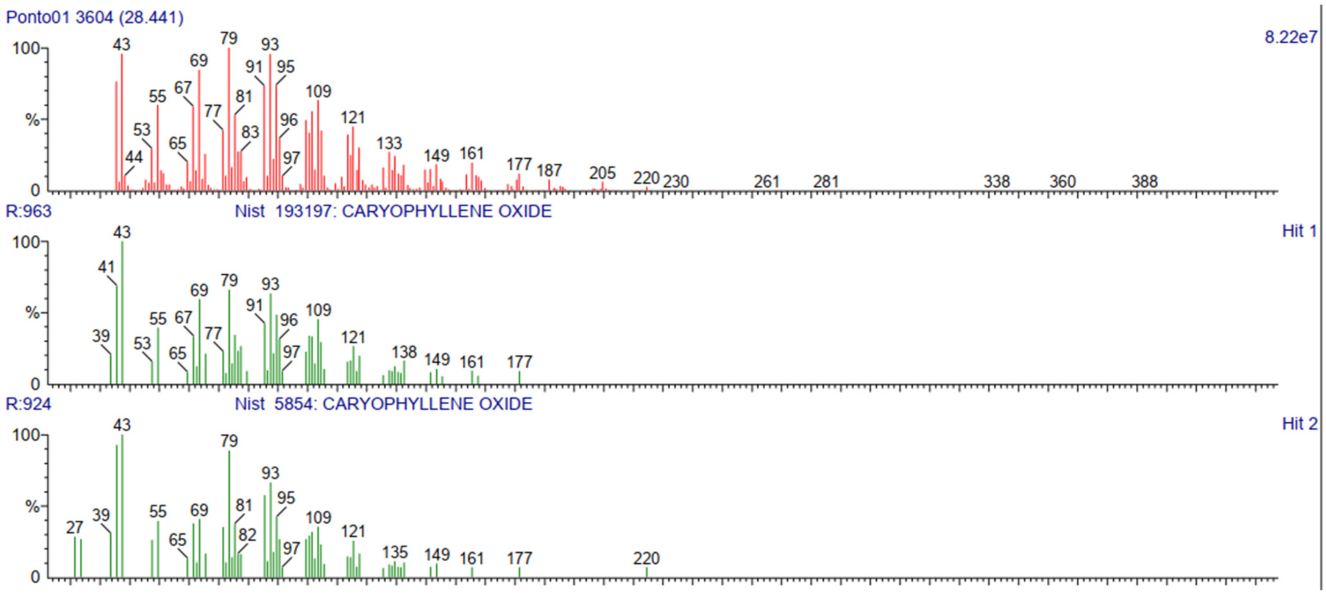

Entry 31: humulene-1,2-epoxide.

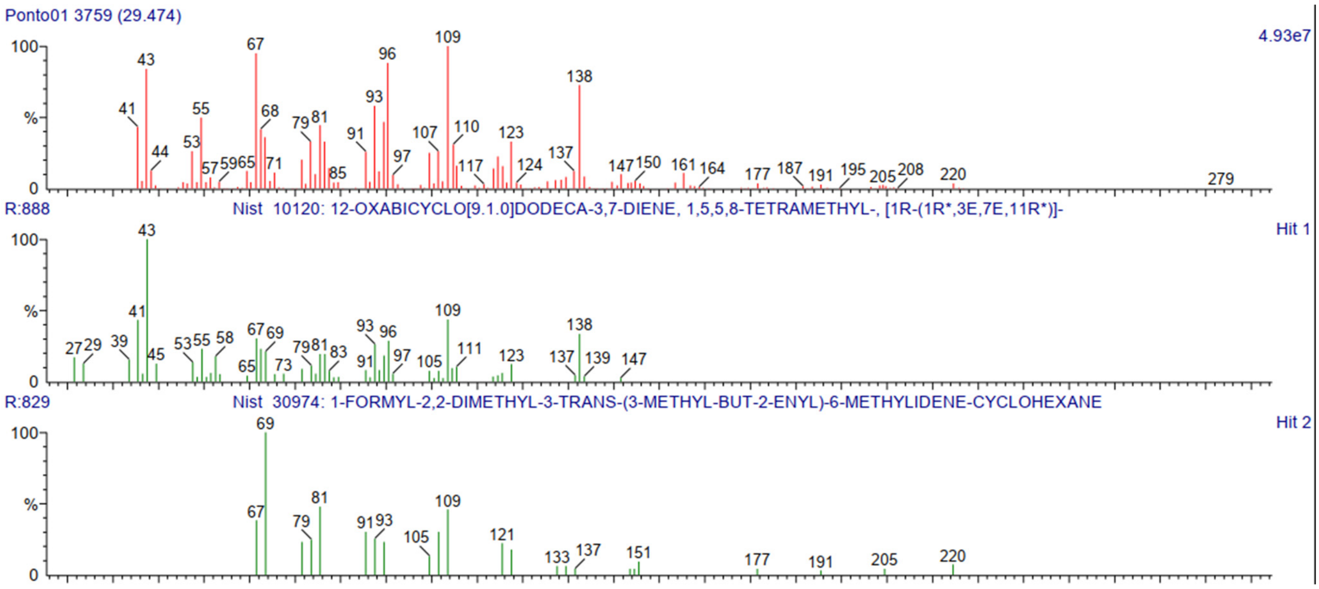

Entry 32: It was assigned as τ-muurolol by the fact that α-cadinol is in entry 33.

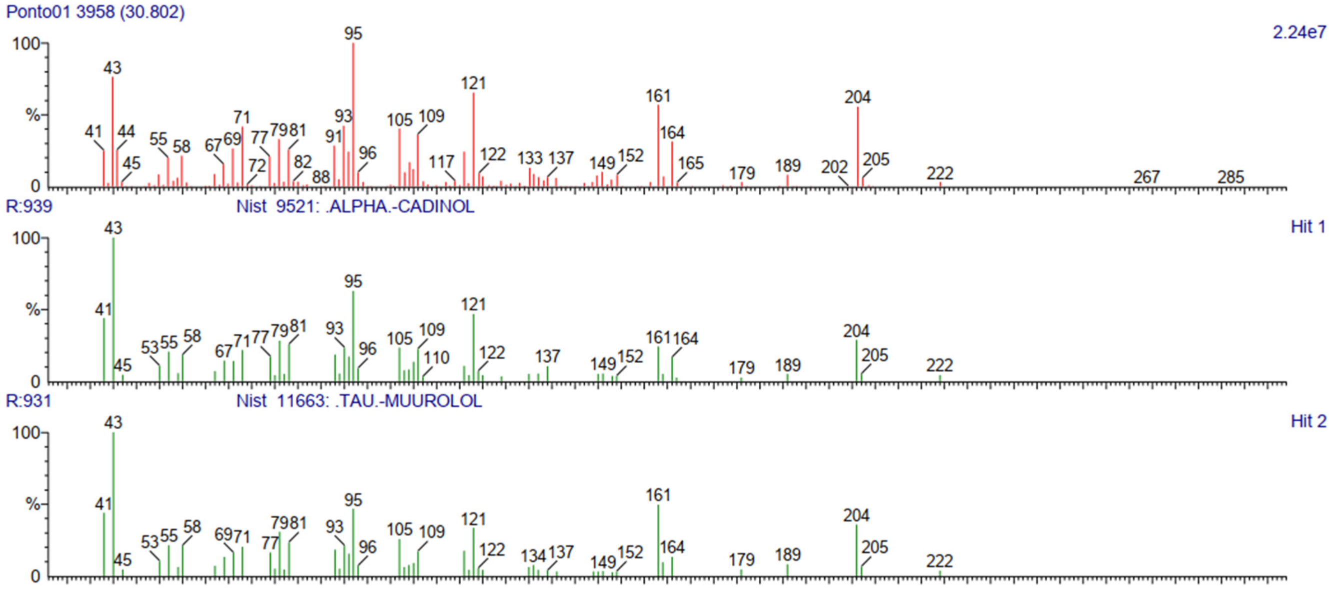

Entry 33: α-muurolol.

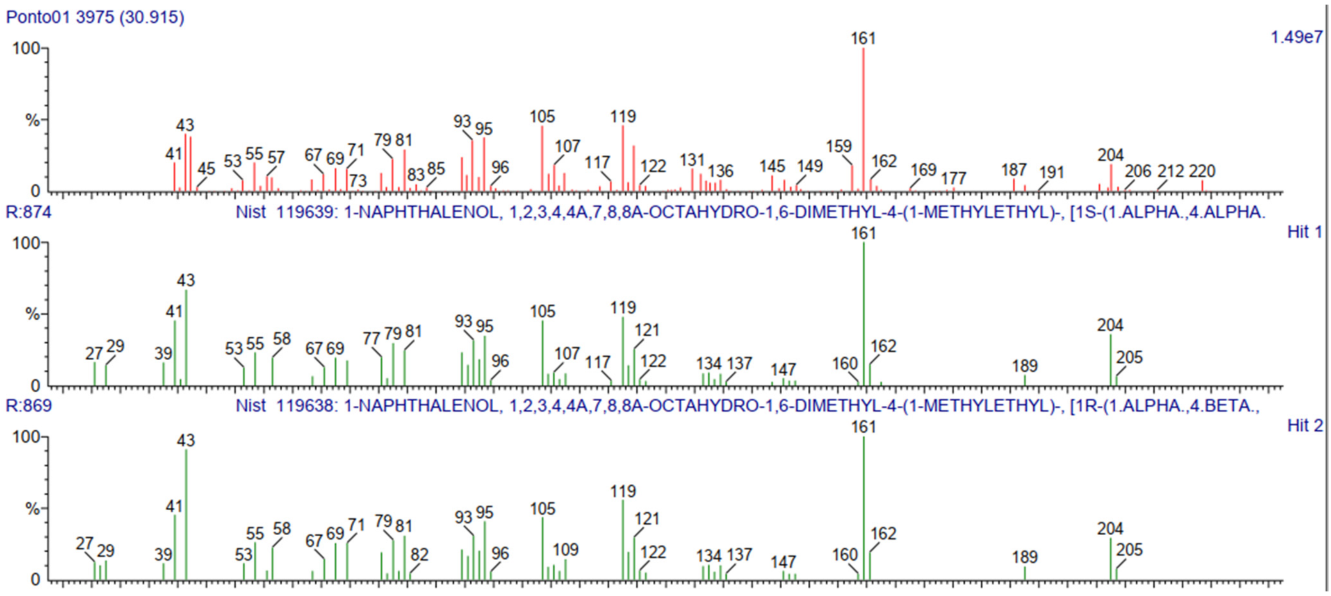

Entry 34: α-cadinol

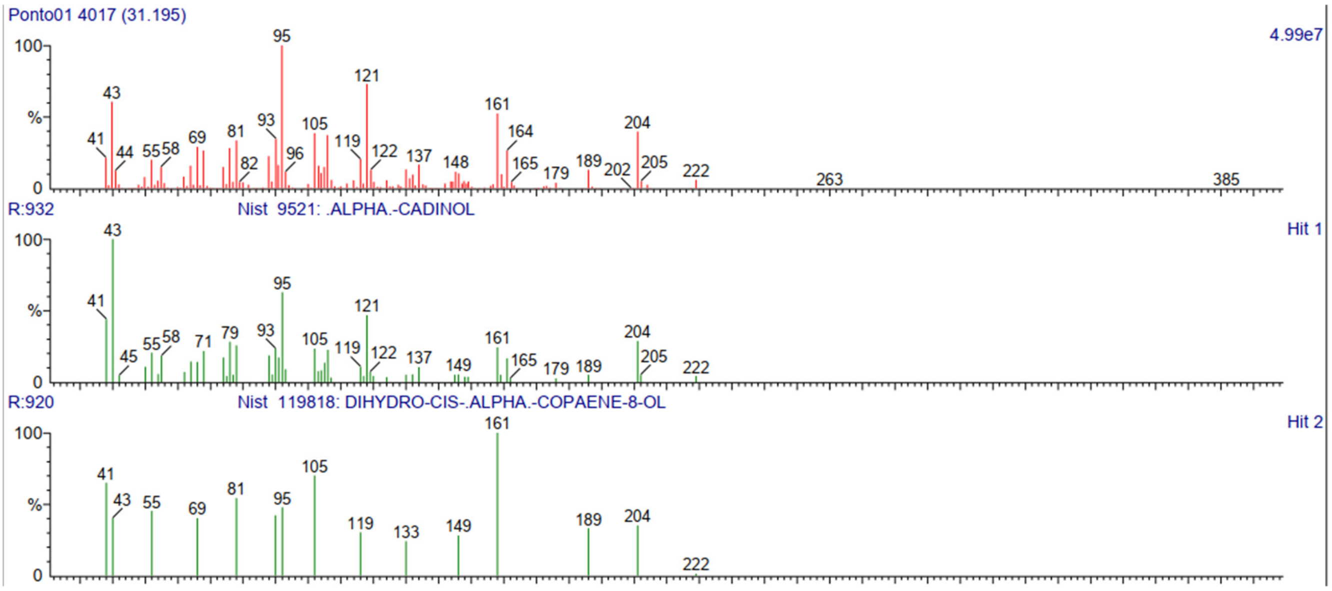

Entry 35: 6*R*,7*R*-bisabolone (AI$_{lit}$ 1740). Retention time 34.38 min.

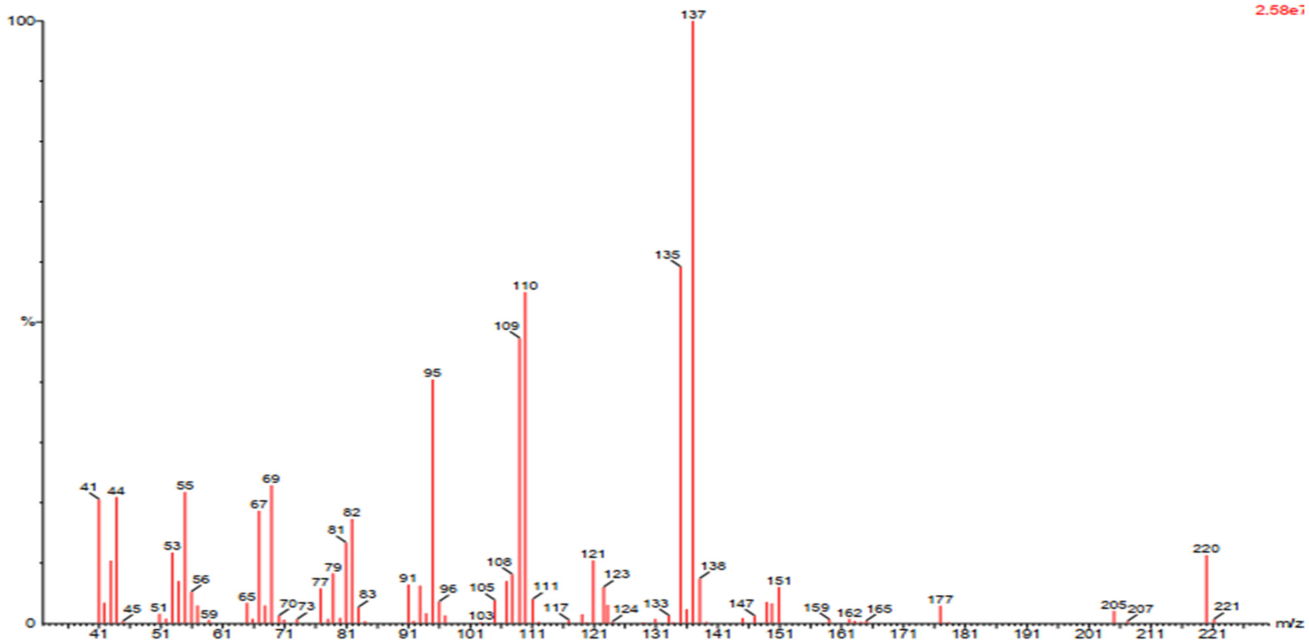

Mass spectrum of constituent with AI$_{exp}$ 1748/Retention time 34.38 min.

RT: 38.24 AI: 1740 KI: 1742 **Bisabolone <(6R,7R)->**
CAS#: 72441-71-5 MF: C15 H24 O FW: 220 MSD LIB#: 138
CN: 2-cyclohexen-1-one, 6-(1,5-dimethyl-4-hexenyl)-3-methyl-,(R-(R*,R*))-
Synonyms: α-oxobisabolene
Source: Amyris, comm. oil, Haiti ex Harold Bussenins; 0.30% *Amyris balsamifera*, Phytochem 28:1909(1989)

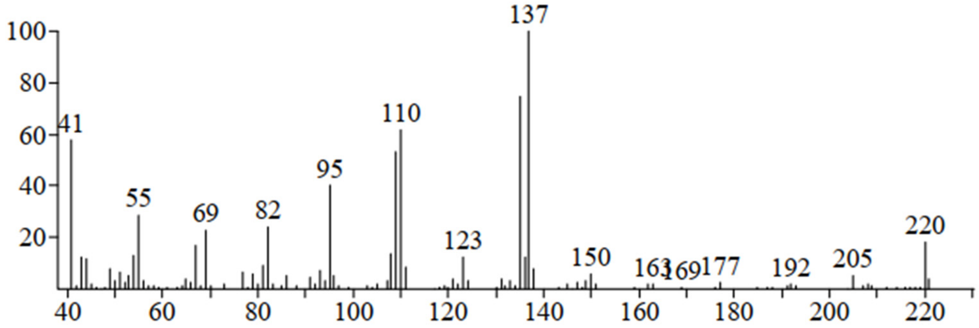

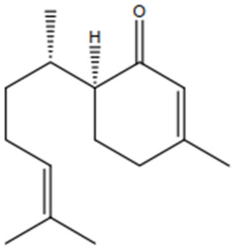

Mass spectrum of 6*R*,7*R*-bisabolone from literature [16].

Entry 36: kaur-16-ene

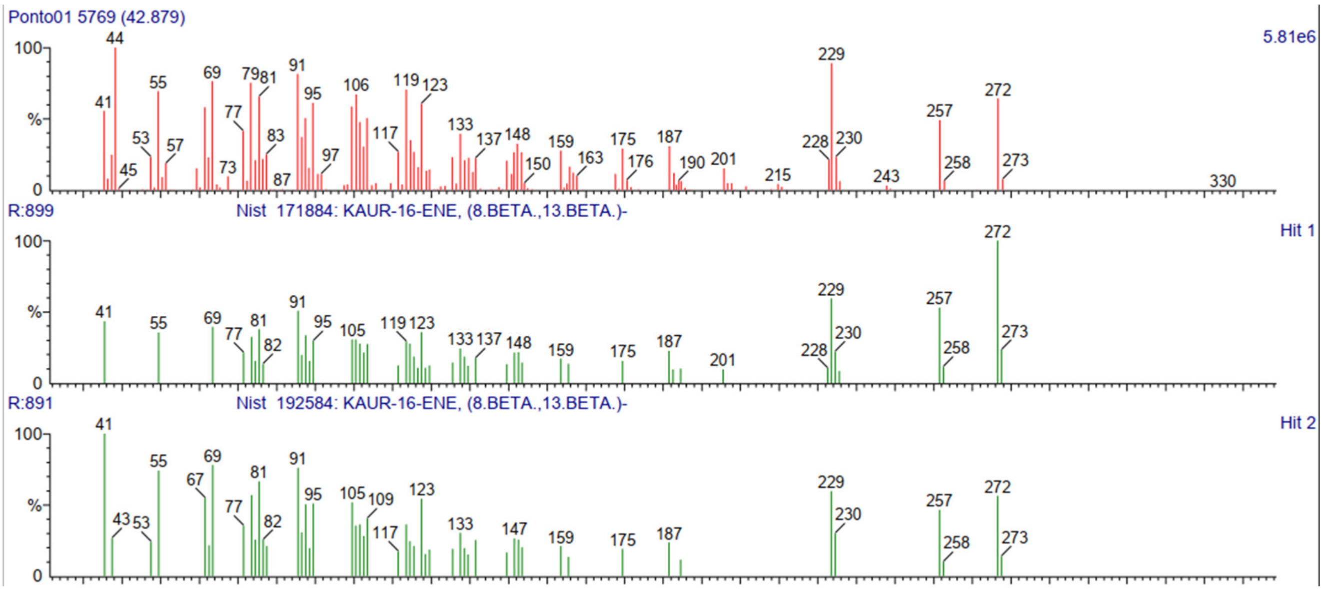

Reference

[16] Adams, R.P. Identification of essential oils components by gas chromatography, mass spectroscopy. 4th ed.; Allured Publishing: Carol Stream, USA, 2017; 809 pp.
